# Age Modifies the Association of Dietary Protein Intake with All-Cause Mortality in Patients with Chronic Kidney Disease

**DOI:** 10.3390/nu10111744

**Published:** 2018-11-13

**Authors:** Daiki Watanabe, Shinji Machida, Naoki Matsumoto, Yugo Shibagaki, Tsutomu Sakurada

**Affiliations:** 1 Department of Pharmacology, St. Marianna University School of Medicine, Kanagawa 216-8511, Japan; d2watanabe@marianna-u.ac.jp (D.W.); matsumoto@marianna-u.ac.jp (N.M.); 2 Division of Nephrology and Hypertension, St. Marianna University School of Medicine, Kanagawa 216-8511, Japan; jnss0322@gmail.com (S.M.); eugo@wc4.so-net.ne.jp (Y.S.)

**Keywords:** chronic kidney disease, protein intake, age, mortality, end-stage renal disease

## Abstract

Whether the effect of a low-protein diet on progression to end-stage renal disease (ESRD) and mortality risk differs between young and elderly adults with chronic kidney disease (CKD) is unclear. We conducted a retrospective CKD cohort study to investigate the association between protein intake and mortality or renal outcomes and whether age affects this association. The cohort comprised 352 patients with stage G3-5 CKD who had been followed up for a median 4.2 years, had undergone educational hospitalization, and for whom baseline protein intake was estimated from 24-h urine samples. We classified the patients into a very low protein intake (VLPI) group (<0.6 g/kg ideal body weight/day), a low protein intake (LPI) group (0.6–0.8 g), and a moderate protein intake (MPI) group (>0.8 g). Compared with the LPI group, the MPI group had a significantly lower risk of all-cause mortality (hazard ratio: 0.29; 95% confidence interval: 0.07 to 0.94) but a similar risk of ESRD, although relatively high protein intake was related to a faster decline in the estimated glomerular filtration rate. When examined per age group, these results were observed only among the elderly patients, suggesting that the association between baseline dietary protein intake and all-cause mortality in patients with CKD is age-dependent.

## 1. Introduction

The number of patients who have end-stage renal disease (ESRD) and are older than 65 years has almost doubled over the past 25 years, and the fastest growing segment of this population in the past decade comprises patients older than 75 years [1,2,3]. In a previously reported large national cohort of patients with chronic kidney disease (CKD) of stage G3 or higher (estimated glomerular filtration rate (eGFR) <60 mL/min/1.73 m^2^) the prognostic implications for death and ESRD varied greatly depending on patient age [4]. Dietary management that ensures energy and protein intake is important for preventing protein-energy wasting, especially in elderly patients [5,6,7,8].

Dietary management for CKD includes a low-protein diet (LPD) of 0.6 to 0.8 g/kg ideal body weight (IBW) per day in patients with stage G3b-5 CKD [9,10,11], partly because it suppresses nitrogen metabolites that cause uremia [12,13]. A meta-analysis of some randomized controlled trials has shown an LPD to be associated with a relatively low risk of composite outcome (renal death and mortality) in patients with non-diabetic CKD [14]. However, results of studies have varied, and some reported studies have been inconclusive [15,16,17]. These previously reported studies involved mainly young rather than elderly patients. Generally speaking, the requirement of protein intake per body weight for elderly persons is higher than that for younger persons [18,19]. Therefore, it might be necessary to modify the level of dietary protein intake according to age rather than to follow an “age neutral” approach in the management of CKD patients, the majority of whom are elderly.

However, to the best of our knowledge, whether the effect of protein intake on either the risk of death or ESRD in the CKD population differs between age groups has not been addressed so far. Therefore, the aim of our study was to investigate the associations between protein intake and mortality or renal outcomes and the interactive effects of age in a cohort of patients with CKD who underwent educational hospitalization.

## 2. Materials and Methods

### 2.1. Study Population

Our study included patients with pre-dialysis CKD of stage G3, G4, or G5 (eGFR < 60 mL/min/1.73 m^2^) who underwent educational hospitalization at St. Marianna University Hospital between 1 January 2011 and 31 December 2016. St. Marianna University Hospital is a tertiary medical care center in Kawasaki city, Kanagawa prefecture, Japan. All CKD patients at our facility are encouraged to undergo educational hospitalization so that they can acquire the necessary knowledge for CKD management and so that medical care providers can communicate with them in situations that are difficult to achieve in an outpatient setting. All patients included in the study were, during the educational hospitalization, advised by a registered dietician to follow a low-protein diet based on the Japanese Society of Nephrology guidelines [11]. This advice was provided to patients individually in a single 20-min session. The target protein intake for patients with CKD stage G3a (eGFR 45 to 59 mL/min/1.73 m^2^) was 0.8 to 1.0 g/kg IBW/day, and that for patients with CKD stage ≥G3b (eGFR < 45 mL/min/1.73 m^2^) was 0.6 to 0.8 g/kg IBW/day [11]. In total, 374 patients with CKD underwent a 1-week educational hospitalization during the time period noted above.

**Figure 1:**
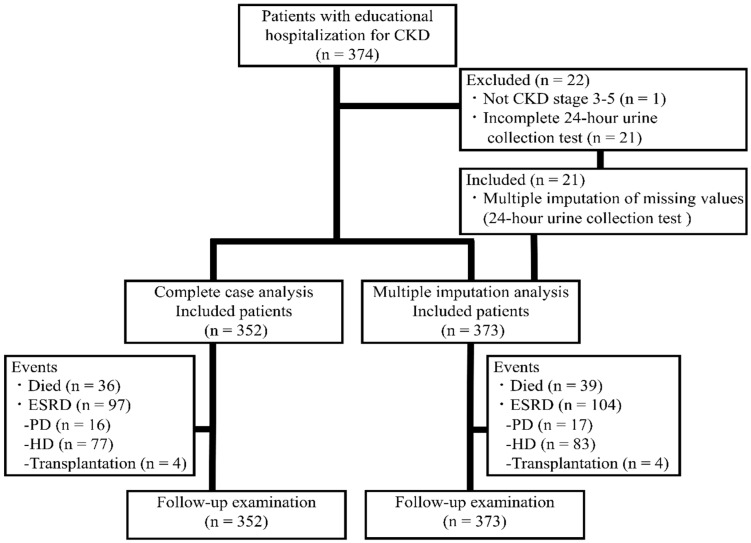
Study flow chart. The study included patients with chronic kidney disease (CKD) admitted for educational hospitalization between 1 January 2011 and 31 December 2016, and they were followed-up until death or 31 December 2017 (open cohort). End-stage renal disease (ESRD) was defined as hemodialysis (HD), peritoneal dialysis (PD), or kidney transplantation. Multiple imputation performed for sensitivity analysis included patients who did not complete the 24-h urine collection (*n* = 21). The complete case analysis and multiple imputation analysis included 352 and 373 patients, respectively.

Of the 374 patients, patients with CKD (ICD-10 codes N18.1-N18.2) stage G1 or G2 (eGFR ≥ 60 mL/min/1.73 m^2^, *n* = 1) and those who did not complete the 24-h urine collection (*n* = 21) were excluded from most of the analyses, leaving 352 patients (Figure 1). The CKD was due to nephrosclerosis (ICD-10 code N26) in 174 patients (49.4%), diabetic nephropathy (ICD-10 code N083) in 120 (34.1%), chronic glomerulonephritis (ICD-10 code N039) in 35 (9.9%), polycystic kidney disease (ICD-10 code Q613) in 8 (2.3%), and other causes in 15 (4.3%). The study protocol was approved by the Ethics Committee of St. Marianna University School of Medicine, Kanagawa, Japan (Approval no. 3855). With this being a retrospective observational study, the need for individual written informed consent was waived. Patients can use the official department website to opt out of the study if they do not want their data used for research purposes. In reporting this study, we have followed the Strengthening the Reporting of Observational Studies in Epidemiology (STROBE) guidelines [20].

### 2.2. Baseline Patient Characteristics and Comorbidities

Baseline clinical and laboratory variables included age, sex, body weight, body mass index (BMI), CKD stage, eGFR, diabetes (ICD-10 codes E10–E14), hypertension (ICD-10 codes I10–I15), cardiovascular disease (CVD), protein intake, sodium intake, alcohol consumption status, smoking status, blood biochemistry (phosphate, albumin, C-reactive protein (CRP), and brain natriuretic peptide (BNP)), drugs (erythropoiesis stimulating agent (ESA), anti-hyperuricemia, phosphate binders, anti-hyperlipidemia, and renin angiotensin system inhibitors (RASi)), systolic blood pressure, left ventricular ejection fraction (LVEF), total-body skeletal muscle mass, urinary protein excretion, and maximum intima-media thickness (max-IMT).

CVD was defined as ischemic heart disease (ICD-10 codes I20–I25), stroke (ICD-10 codes I60–I67), or congestive heart failure (ICD-10 I50). Data were extracted from patients’ electronic medical records by researchers other than the researcher who performed the statistical analysis. Total body skeletal muscle mass was estimated from 24-h urinary creatinine excretion by the Wang et al. equation [21], and eGFR was calculated by means of the Modification of Diet in Renal Disease (MDRD) Study equation for Japanese patients with CKD [22].

### 2.3. Endpoints

The study endpoints were mortality and ESRD. Survival and ESRD were determined from the day of initial examination until 31 December 2017, and the median follow-up period was 4.2 (range: 0.6 to 6.9) years. ESRD was defined as CKD requiring hemodialysis, peritoneal dialysis, or kidney transplantation. No patient was lost to follow-up. To exclude the possibility that death or ESRD occurring early in the follow-up period was due to an undiagnosed preclinical disease at the time, baseline assessment was done (reverse causation), which may also have been related to our exposure, and we also examined the association between protein intake, death and ESRD after the exclusion of events that were recorded within the first 6 months of follow-up (death (*n* = 6) and ESRD (*n* = 25)). A secondary outcome was defined as mean change per year in renal function (eGFR or spot urine protein value), body weight, or in serum albumin or phosphate concentration.

### 2.4. Dietary Protein Intake

Dietary protein intake was estimated by the Maroni et al. equation on the basis of a least one 24-h urine sample [23] and calculated as follows: protein intake = 6.25 × (urinary nitrogen excretion (g/24 h)) + weight (kg) × 0.031 (g/kg/day)). We believe the Maroni et al. equation to be valid because non-urea nitrogen excretion values obtained by this equation match values obtained by another reported equation [24]. During the educational hospitalization period, 24-h urine collection was performed twice—once on the day of admission and once on the day following the 1-day home-stay break. Dietary protein intake has been reported to have greater within-individual variation than between-individual variation [25]. However, the reported protein intake was evaluated from dietary records of community-dwelling persons; estimation of protein intake from 24-h urine samples from CKD patients has been limited. We estimated the number of days and group size required to ensure accuracy in estimating the usual (“true”) mean intake as well as within- and between-patient variations in dietary protein and sodium intake based on 24-h urine samples [25]. To obtain an optimally accurate estimation of habitual protein intake, we used the mean protein intake estimated from two different 24-h urine samples. Protein intake was calculated as g/kg IBW/day. We used the sample mean X to make statistical inference regarding the population mean μ.

### 2.5. Statistical Analysis

Values are presented as mean with standard deviation (SD) and 95% confidence interval (CI), median with interquartile range, or number with percentage. Continuous variables were compared by analysis of variance (ANOVA) and Kruskal–Wallis test. Categorical variables were compared by chi-square test. Absolute risk for mortality or ESRD is shown as event per 1000 patient-years.

Guidelines issued by the Japanese Society of Nephrology recommend a protein intake of 0.6 to 0.8 g/kg IBW/day for patients with CKD of stage G3b-5. We divided the study patients into three groups: a very low protein intake (VLPI) group (<0.6 g/kg IBW/day), a low protein intake (LPI) group (0.6 to 0.8 g/kg IBW/day), and a moderate protein intake (MPI) group (>0.8 g/kg IBW/day). Multivariate Cox proportional hazard regression and Fine and Gray (sub-distribution hazards models) analyses were performed to adjust for the effects of several baseline clinical variables and cardiovascular risk factors [26]. To avoid multicollinearity, correlation analyses were performed among variables in each model based on phi coefficient for two nominal variables, correlation ratio for one nominal and one continuous variable, and Spearman’s correlation coefficient for two continuous variables. In the multivariate model, the covariates included age, sex, BMI, CKD stage, diabetes, prior CVD, sodium intake, alcohol consumption status, smoking status, blood biochemistry (serum phosphate, albumin, CRP, and BNP), drugs (ESA, anti-hyperuricemia, phosphate binders, anti-hyperlipidemia, and RASi), systolic blood pressure, LVEF, total-body skeletal muscle mass, urinary protein excretion, and max-IMT. Hazard ratios (HRs) for mortality and for ESRD are presented as HR (95% CI), with the LPI group as the reference. To estimate the association between events attributed to protein intake and age, we also examined the relation between protein intake and events per age group (≤65 years and >65 years) [27]. Data on secondary outcomes were obtained from the yearly change in eGFR, spot urine protein, body weight, and serum albumin and phosphate as calculated by the slope of a linear regression line fitted using least squares regression. To investigate the association between protein intake and change in secondary outcomes, the same adjustment factors noted above were used.

Patients for whom information was missing pertaining to the level of urinary protein excretion, sodium and protein intake, and skeletal muscle mass (5 men and 16 women) were excluded from the main analyses. Missing data for most of these patients was due to the failure to collect the full 24-h urine sample. Such missing data could lead to a systematic error appearing as a selection bias. For sensitivity analysis, we performed multiple imputation on five data sets using multivariate imputation by chained equation (MICE) from R statistical software [28]. Missing data were assumed to be missing at random. A *p* value < 0.05 for two-sided tests was considered significant. The linear trend was computed by treating exposure as a continuous variable. HRs and the corresponding 95% CIs were estimated for a 0.1 g/kg IBW/day increase in protein intake. The statistical analyses were performed with the use of R software 3.4.3 (R Core Team, Vienna, Austria) and/or JMP Pro for Windows (SAS institute Inc., Cary, NC, USA), and the researcher who performed the statistical analysis was blinded to the dietary guidance given to the patients.

## 3. Results

### 3.1. Baseline Characteristics of the Cohort

Baseline clinical characteristics of the study patients are shown in Table 1. Mean age, proportion of males, and median eGFR of the 352 CKD patients were 70.2 (range: 20 to 91) years, 29.0%, and 22.9 (range: 4.7 to 57.0) mL/min/1.73 m^2^, respectively. Three hundred fifteen patients (89.5%) had hypertension, and most of these patients were taking an antihypertensive medication such as angiotensin-converting enzyme inhibitor, angiotensin receptor blocker, and/or calcium channel blocker. One hundred thirty patients (36.9%) were being treated with ESA. Most patients were treated with other drugs commonly used in CKD, such as phosphate and potassium binders and diuretics. A low BMI (<18.5 kg/m^2^) was found in 9 patients (12.0%), 8 patients (5.0%), and 1 patient (0.9%) in the VLPI, LPI, and MPI groups, respectively, and obesity (≥25.0 kg/m^2^) was found in 20 (26.7%), 51 (31.7%), and 72 (62.0%) patients in the VLPI, LPI, and MPI groups, respectively.

No patient was taking oral amino acid supplementation. Of the patients included in the study, 290 (82.4 %) completed the 24-h urine collection twice during the educational hospitalization period (the other 62 patients completed it only once). Protein intake estimated from 24-h urine samples did not differ between the first and second samples (Table S1). Because between-patient variation in protein intake is greater than within-patient variation among CKD patients, we surmised that we could determine the groups’ and individual patients’ usual (“true”) mean intake and individual ranking from the two 24-h urine samples we obtained from patients (Table S2). The mean protein intake was 42.0 (range: 18.7 to 83.0) g/day in the 352 patients. The numbers of patients with VLPI (range: 18.7 to 44.8 g/day), LPI (range: 28.5 to 55.1 g/day), and MPI (range: 35.2 to 83.0 g/day) were 75, 161, and 116, respectively.

### 3.2. Protein Intake and Endpoints

**Figure 2:**
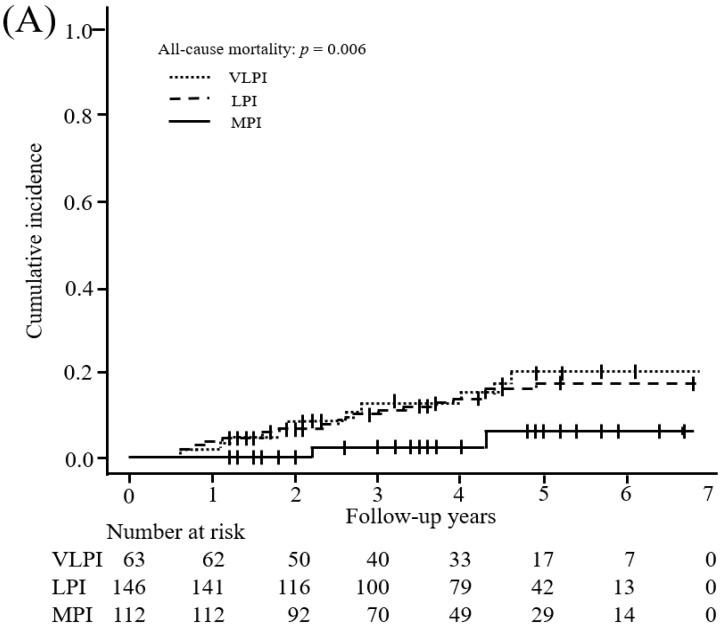

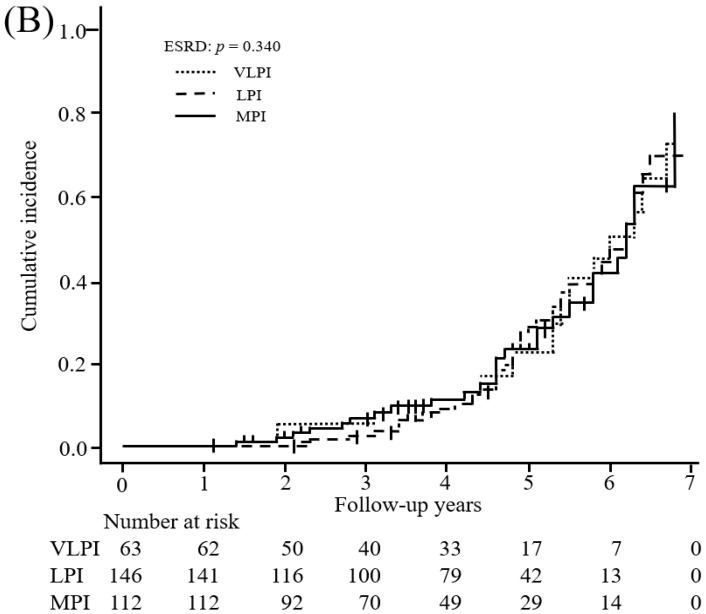
Competing risk model for association between protein intake and the study endpoints. Cumulative incidences (95% confidence interval) of (**A**) mortality and (**B**) end-stage renal disease (ESRD) in the very low protein intake (VLPI), low protein intake (LPI), and moderate protein intake (MPI) groups were 0.199 (0.099 to 0.324), 0.170 (0.107 to 0.246), and 0.060 (0.018 to 0.138), respectively, and 0.801 (0.513 to 0.929), 0.695 (0.519 to 0.818), and 0.781 (0.259 to 0.956), respectively. Thirty-one patients were excluded because of death (*n* = 6) or development of ESRD (*n* = 25) within the first 6 months of follow-up. ESRD was defined as hemodialysis, peritoneal dialysis, or kidney transplantation. Multivariable models included age, sex, BMI, CKD stage, and comorbidities (DM, CVD, and anemia) (Model 1).

During the follow-up period, 36 patients died, including 10 patients (13.3%) in the VLPI group, 22 (13.7%) in the LPI group, and 4 (3.4%) in the MPI group (Table 2). The incidence of death per 1000 patient-years decreased as protein intake increased. We showed an inverse association between protein intake and all-cause mortality among all patients (VLPI: HR, 1.42 (95% CI: 0.55 to 3.44); LPI: reference; MPI: HR, 0.29 (95% CI: 0.07 to 0.94); *p* value < 0.001) with adjustment for potential confounders (Model 2). Moreover, the multivariable-adjusted HR (95% CI) of all-cause mortality for a 0.1 g/kg IBW/day increment was 0.76 (0.60 to 0.95). In the subgroup analysis, elderly patients (age > 65 years) showed a significantly inverse association between protein intake and all-cause mortality (VLPI: HR, 1.52 (95% CI: 0.51 to 4.27); LPI: reference; MPI: HR, 0.14 (95% CI: 0.02 to 0.69); *p* value < 0.001), whereas younger patients (age ≤ 65 years) showed no significant association with death (VLPI: HR, 2.54 (95% CI: 0.09 to 70.13); LPI: reference; MPI: HR, 3.73 (95% CI: 0.31 to 94.60); *p* value = 0.879). A competing-risk model (Figure 2) and multiple imputation modeling as sensitivity analysis (Model 3) showed results similar to those in the multivariable-adjusted model (Model 2).

Ninety-seven patients developed ESRD, including 22 patients (29.3%) in the VLPI group, 48 (29.8%) in the LPI group, and 27 (23.3%) in the MPI group, during the follow-up period (Table 2). The incidence of ESRD per 1000 patient-years decreased as protein intake increased. However, the multivariable-adjusted model (VLPI: HR, 0.87 (95% CI: 0.49 to 1.56); LPI: reference; MPI: HR, 0.72 (95% CI: 0.40 to 1.29); *p* value = 0.631) and multiple imputation model (VLPI: HR, 0.83 (95% CI: 0.47 to 1.47); LPI: reference; MPI: HR, 0.62 (95% CI: 0.35 to 1.09); *p* value = 0.722) showed that the association of protein intake with the risk of ESRD was not statistically significant.

### 3.3. Protein Intake and Secondary Outcomes

The yearly changes in renal function and other outcomes are shown in Table 3. Protein intake was related with a yearly decline of eGFR (0.84 (95% CI: −3.69 to 5.37) in the VLPI group, −0.23 (95% CI: −4.80 to 4.34) in the LPI group, and −0.97 (95% CI: −5.63 to 3.70) mL/min 1.73 m^2^/year in the MPI group; *p* value = 0.032), whereas it was not significantly associated with spot urine protein, body weight, or serum albumin and phosphate. In the subgroup analysis, elderly patients showed a significant inverse association between protein intake and yearly change in eGFR (1.06 (95% CI: −4.24 to 6.36) in the VLPI group, −0.69 (95% CI: −6.02 to 4.63) in the LPI group, and −0.98 (95% CI: −6.46 to 4.49) mL/min 1.73 m^2^/year in the MPI group; *p* value = 0.028).

## 4. Discussion

We aimed to investigate the associations between protein intake and mortality or renal outcomes in cohort patients with CKD who underwent educational hospitalization. We found that moderate dietary protein intake, which is even higher than that recommended in the Japanese CKD guidelines, was in fact associated with lower all-cause mortality compared with low protein intake (corresponding to that recommended by the Japanese guidelines) or very low protein intake in patients with CKD, especially in the elderly. It is also intriguing that this survival benefit was achieved without compromising kidney prognosis. To the best of our knowledge, this is the first longitudinal cohort study to show that dietary protein intake affects mortality in these patients.

Although a meta-analysis including data from randomized clinical trials has reported that LPD decreased the risk of composite outcomes (mortality and renal death) in non-diabetic CKD patients [14], this finding has not been reproduced in studies in patients with pre-dialysis CKD, who are recommended to restrict protein intake [15,16]. One recent study found that LPD decreased only the risk of ESRD but not mortality [29]. Another study showed higher or lower than estimated protein intake from normalized protein nitrogen appearance to be associated with higher risk of mortality compared with the reference range of normalized protein nitrogen appearance (0.90 to 0.99 g/kg/day), but this study population did not comprise patients with pre-dialysis CKD but rather those undergoing maintenance hemodialysis [30]. Some epidemiological and clinical studies have investigated the association between protein intake and mortality in younger patients (≤65 years) [14,29], but such studies in elderly patients (>65 years) have been scarce. Levine et al. reported that high and moderate protein intakes were associated with higher risk of all-cause and cancer mortality in participants ≤65 years old but that they decreased these mortalities in those >65 years old [27]. It was speculated that the restriction in protein intake caused low energy intake, negative nitrogen balance, and low insulin-like growth factor-1 in patients >65 years old [31]. From a meta-analysis of nitrogen balance studies, the recommended dietary allowance for estimated protein intake at the 97.5th percentile of the acceptable range of intake for healthy adults was found to be 0.83 g/kg/day [18]. In elderly people (56 to 80 years old) in general, the recommendation for protein intake is estimated to be 1.0 to 1.25 g/kg/day from current and retrospective nitrogen balance data [19]. Therefore, this recommended protein intake according to the guidelines may result in a risk of malnutrition and low muscle mass and strength from insufficient dietary intake because the requirement for protein intake per weight is higher in elderly persons than in younger adults [32,33]. One recent study reported that elderly CKD patients have an increased risk of death before reaching ESRD, although this chronological relation is reversed in middle-age patients [4]. One reason why the high risk of mortality rather than that of ESRD became more pronounced in the present study might be that it included a high proportion of patients of advanced age. These findings should alert us to consider the balance between the positive and negative effects of protein restriction according to patient age; i.e., prescribing a protein intake adequate for maintaining energy and nitrogen equilibrium rather than prescribing a uniform LPD aimed at preventing ESRD may contribute to overall patient benefit by reducing the risk of mortality especially in elderly patients with CKD.

The present study showed that the higher the protein intake, the faster the decline of eGFR, whereas this relation of protein intake was not evident with proteinuria assessed by spot urine protein, body weight, and serum albumin and phosphate. In addition, protein intake was not related to the risk of ESRD. Previous studies have produced controversial results with regard to protein intake and renal outcomes [34,35]. These controversial results may be due to differences in study design, method and amount of protein intake, primary disease (such as diabetes or nephrosclerosis), and the observation periods. One meta-analysis reported that an LPD has an inverse association with ESRD risk [14]. However, most of the analyzed studies reported that protein intake (0.3 to 0.6 g/kg/day vs. >0.6 g/kg/day) was not associated with ESRD risk. Therefore, the preventive effect of an LPD on ESRD may be small. Recently, a proportional decline in GFR and increase in ESRD risk was reported [36,37]. The National Kidney Foundation and U.S. Food and Drug Administration working group reported that a decline of 10% in eGFR was related to the risk of ESRD [36], but this was not reproduced in the Japanese CKD cohort study (CKD-JAC) [37]. The present study showed that the % per year change in eGFR in the VLPI, LPI, and MPI groups was 4.1 (from 19.5 at baseline to 20.3 mL/min/1.73 m^2^ after 1 year), −0.9 (from 21.8 at baseline to 21.6 mL/min/1.73 m^2^ after 1 year), and −3.7 (from baseline 26.7 to 25.7 mL/min/1.73 m^2^ after 1 year), respectively. Protein intake may be related to the risk of ESRD when a larger sample size and longer observation period than that in the present study are evaluated. This again suggests the importance of balancing the risk of mortality and that of ESRD according to patient characteristics (mortality carries more weight in elderly, frail patients).

Several limitations to the present study should be noted. First, the study was retrospective, and despite the multivariate analysis, we were not able to control for all potential confounding factors, particularly frailty-related factors. We acknowledge that some baseline patient characteristics varied between groups. Second, protein intake was evaluated only during educational hospitalization. This might have led to misclassification bias because educational hospitalization might have modified a patient’s usual protein intake. In addition, the MPI group included patients with a maximum protein intake of 1.35 g/kg IBW/day, meaning that protein intake was not actually moderate in all patients in this group. Therefore, further research is needed to establish optimal dietary protein intake levels for CKD patients. However, we evaluated protein intake in most of the patients twice rather than once to avoid such bias. In addition, protein intake estimated from this study varied more between patients than per patient (Table S1), which indicates that our sample size and the number of measures were sufficient (Table S2). Third, the study included only patients who consented to participate in the educational hospitalization program. Such patients might be more health conscious compared with those who did not. Fourth, the follow-up period was relatively short, and this might have an influence on the association between protein intake and risk of ESRD. The difference between absolute (event/patient years) and cumulative risk might not only be the sub-distribution hazard but also the effect of censoring data due to the relatively short follow-up (Table 2, Figure 2). Our analysis considered only two competing events (all-cause mortality and ESRD); therefore, deaths occurring after the development of ESRD were not considered in this analysis. Moreover, the number of deaths was relatively low, and we could not conduct an analysis of each cause of death. Fifth, there was no dietary survey, so energy and non-protein nutrition intakes could not be evaluated. We could not exclude the confounding factor that protein intake has a strong positive correlation with energy and some non-protein nutrition intake. Energy intake has been reported to be inversely associated with all-cause mortality and vascular mortality in persons with and without reduced kidney function [38]. So that the effect of protein intake on the prevention of mortality would not be overestimated, we focused on change in body weight per year, which is an indicator of energy balance [39,40]. Change in body weight is one of five important indicators of malnutrition [39], and it reflects energy intake [40]. There was no difference in body weight change between the three protein intake-based groups (Table 3). Thus, there was no between-group difference in energy intake required by the patients, and our results suggest that protein intake is also important, not simply a result reflecting insufficient energy intake alone. A previous study reported that animal protein derived from red meat, but not vegetable protein, was associated with an increased risk of ESRD [41]. However, we could not differentiate animal from vegetable protein because the present study estimated habitual protein intake from 24-h urine samples. Therefore, a well-designed multicenter study that further evaluates energy and nutrient intake via a dietary survey is needed.

## 5. Conclusions

Our findings suggest that dietary protein intake of >0.8 g/kg IBW/day, a consumption level that is higher than the guidelines-recommended level, is an age-dependent risk factor for all-cause mortality in patients with CKD.

## Figures and Tables

**Table 1:** Baseline characteristics of the study patients with CKD ^1,2^ per protein intake.

	All	Protein Intake			*p* Value ^3^
		Very Low	Low	Moderate	
	(*n* = 352)	(*n* = 75)	(*n* = 161)	(*n* = 116)	
Age (years)	70.2 ± 11.4	70.6 ± 11.9	71.2 ± 11.0	68.6 ± 11.5	0.173
≤65 (*n* (%))	93 (26.4)	16 (21.3)	39 (24.2)	38 (32.8)	0.150
>65 (*n* (%))	259 (73.6)	59 (78.7)	122 (75.8)	78 (67.2)	
Male (*n* (%))	102 (29.0)	24 (32.0)	49 (30.4)	29 (25.0)	0.499
Smoking (*n* (%))	58 (16.5)	11 (14.7)	26 (16.2)	21 (18.1)	0.614
Body weight (kg)	64.2 ± 13.6	60.6 ± 13.2	62.0 ± 12.3	69.6 ± 15.4	**<0.001**
BMI (kg/m^2^) ^4^	24.7 ± 4.4	23.1 ± 4.1	24.0 ± 3.5	26.9 ± 4.9	**<0.001**
Skeletal muscle mass (kg) ^5,6^	19.3 ± 6.5	14.7 ± 5.8	18.6 ± 5.1	23.2 ± 6.4	**<0.001**
CKD stage (*n* (%))					
3a	20 (5.7)	2 (2.7)	6 (3.7)	12 (10.3)	**<0.001**
3b	72 (20.5)	13 (17.3)	29 (18.0)	30 (25.9)	
4	149 (42.3)	23 (30.7)	76 (47.2)	50 (43.1)	
5	111 (31.5)	37 (49.3)	50 (31.1)	24 (20.7)	
eGFR (mL/min/1.73 m^2^) ^7^	22.9 ± 11.3	19.5 ± 11.0	21.8 ± 10.2	26.7 ± 12.0	**<0.001**
UPE (g/24 h) ^6^	0.81 (0.18 to 2.00)	1.2 (0.26 to 2.61)	0.73 (0.19 to 1.77)	0.72 (0.16 to 1.83)	0.089
Serum phosphate (mg/dL)	3.7 ± 0.8	3.9 ± 1.0	3.7 ± 0.7	3.7 ± 0.7	**0.030**
Hypertension history (*n* (%))	315 (89.5)	67 (89.3)	145 (90.1)	103 (88.8)	0.943
CVD history (*n* (%))	151 (42.9)	34 (45.3)	75 (46.6)	42 (36.2)	0.204
Systolic blood pressure (mmHg)	135 ± 21	137 ± 21	132 ± 21	137 ± 19	0.161
LVEF (%)	64.0 ± 10.6	63.8 ± 11.2	62.9 ± 10.9	65.6 ± 9.4	0.119
DM history (*n* (%))	157 (44.6)	38 (50.7)	69 (42.9)	50 (43.1)	0.492
Anemia (*n* (%))	177 (50.3)	45 (60.0)	85 (52.8)	47 (40.5)	**0.021**
Serum albumin (g/dL)	3.9 ± 0.4	3.7 ± 0.4	3.9 ± 0.5	4.0 ± 0.4	**<0.001**
CRP (mg/dL)	0.09 (0.04 to 0.22)	0.10 (0.03 to 0.35)	0.07 (0.04 to 0.18)	0.10 (0.04 to 0.20)	0.330
BNP (pg/mL)	90 (39 to 221)	132 (66 to 238)	89 (42 to 224)	62 (29 to 182)	**0.002**
Protein intake (g/day) ^6,8^	42.0 ± 10.7	30.5 ± 5.6	39.7 ± 5.9	52.7 ± 8.3	**<0.001**
Protein intake (g/kg IBW/day) ^6,8^	0.74 ± 0.08	0.52 ± 0.07	0.70 ± 0.06	0.93 ± 0.10	**<0.001**
Sodium intake (mmol/day) ^6^	62.9 ± 21.4	57.4 ± 21.3	61.7 ± 19.6	68.0 ± 22.7	**0.002**

^1^ Continuous and categorical values are shown as mean ± standard deviation (SD), median (interquartile range), or number (percentage). Bold values are statistically significant (*p* < 0.05). BMI, body mass index; BNP, brain natriuretic peptide; CKD, chronic kidney disease; CRP, C-reactive protein; CVD, cardiovascular disease; DM, diabetes mellitus; eGFR, estimated glomerular filtration rate; IBW, ideal body weight; LVEF, left ventricular ejection fraction; UPE, urinary protein excretion. ^2^ Ideal body weight was defined as a body mass index of 22 kg/m^2^. ^3^ Analysis of variance (ANOVA), Kruskal–Wallis test, or chi-square test was used to evaluate distribution. ^4^ The body mass index is the weight in kilograms divided by the square of the height in meters. ^5^ Total-body skeletal muscle mass = 21.8 × urinary creatinine excretion (g/24 h). ^6^ To reduce random error, mean values were calculated on the basis of two 24-h urine samples. ^7^ eGFR (mL/min/1.73 m^2^) = 194 × serum creatinine (−1.094) × age (−0.287) × 0.739 (if female). ^8^ Protein intake = 6.25 × (urinary nitrogen excretion (g/24 h) + weight (kg) × 0.031 (g/kg/day)).

**Table 2:** Multivariable-adjusted hazard ratios (HRs) and 95% confidence intervals for mortality according to dietary protein intake ^1^.

	VLPI		LPI		MPI		0.1 g/kg IBW/day Increment		*p* Value ^2^
	**0.29 to 0.59** **(g/kg IBW/day)**		**0.60 to 0.80** **(g/kg IBW/day)**		**0.81 to 1.35** **(g/kg IBW/day)**				
Mean (SD) protein intake (g/kg IBW/day)	0.52 (0.07)		0.70 (0.06)		0.93 (0.10)		―		―
**All-cause mortality**									
No of deaths/patient years	10/284.9		22/613.3		4/426.6		―		―
Rate/1000 patient years	35.1	(23.7 to 46.5)	35.9	(24.4 to 47.4)	9.4	(3.4 to 15.4)	―		―
All									
Model 1 ^3^	0.89	(0.39 to 1.88)		1.00 (ref)	0.29	(0.08 to 0.77)	0.89	(0.80 to 0.99)	**<0.001**
Model 2 ^4^	1.42	(0.55 to 3.44)		1.00 (ref)	0.29	(0.07 to 0.94)	0.76	(0.60 to 0.95)	**<0.001**
Model 3 ^5^	1.45	(0.57 to 3.48)		1.00 (ref)	0.27	(0.07 to 0.81)	0.83	(0.70 to 0.98)	**<0.001**
≤65 years ^6^									
Model 1 ^3^	2.54	(0.09 to 70.13)		1.00 (ref)	3.73	(0.31 to 94.60)	1.00	(0.01 to 82.07)	0.879
Model 2 ^4^	―			―	―		―		―
Model 3 ^5^	―			―	―		―		―
>65 years ^6^									
Model 1 ^3^	0.84	(0.35 to 1.85)		1.00 (ref)	0.16	(0.03 to 0.55)	0.57	(0.49 to 0.67)	**<0.001**
Model 2 ^4^	1.52	(0.51 to 4.27)		1.00 (ref)	0.14	(0.02 to 0.69)	0.65	(0.47 to 0.89)	**<0.001**
Model 3 ^5^	1.64	(0.58 to 4.45)		1.00 (ref)	0.14	(0.02 to 0.59)	0.69	(0.51 to 0.93)	**<0.003**
**ESRD** ^7^									
No. of events/patient years	22/171.6		48/436.0		27/340.8		―		―
Rate/1000 patient years	128	(107 to 149)	110	(90 to 129)	79	(63 to 96)	―		―
All									
Model 1 ^3^	0.97	(0.58 to 1.60)		1.00 (ref)	0.70	(0.43 to 1.16)	1.00	(0.21 to 4.85)	0.689
Model 2 ^4^	0.87	(0.49 to 1.56)		1.00 (ref)	0.72	(0.40 to 1.29)	1.00	(0.54 to 1.88)	0.631
Model 3 ^5^	0.83	(0.47 to 1.47)		1.00 (ref)	0.62	(0.35 to 1.09)	1.00	(0.62 to 1.64)	0.722
≤65 years ^6^									
Model 1 ^3^	1.20	(0.43 to 2.93)		1.00 (ref)	0.71	(0.28 to 1.69)	1.00	(0.01 to 81.05)	0.508
Model 2 ^4^	8.31	(0.78 to 88.42)		1.00 (ref)	0.92	(0.14 to 6.07)	1.01	(0.21 to 4.83)	**0.039**
Model 3 ^5^	4.12	(0.47 to 36.26)		1.00 (ref)	0.78	(0.14 to 4.22)	1.01	(0.24 to 4.31)	0.054
>65 years ^6^									
Model 1 ^3^	0.80	(0.42 to 1.47)		1.00 (ref)	0.85	(0.45 to 1.57)	1.00	(0.17 to 5.87)	0.719
Model 2 ^4^	0.46	(0.20 to 1.06)		1.00 (ref)	0.60	(0.27 to 1.37)	1.00	(0.49 to 2.04)	0.778
Model 3 ^5^	0.47	(0.21 to 1.03)		1.00 (ref)	0.70	(0.33 to 1.48)	1.00	(0.49 to 2.06)	0.757

^1^ All values are mean (SD), number and relative hazards (95% confidence interval). All estimates were derived from multivariable Cox proportional hazards regression models. Bold values are statistically significant (*p* < 0.05). “―” signs not calculated the statistic values in the table. ESRD, end-stage renal disease. ^2^ *p*-linear trend was calculated by using the Wald test statistic. ^3^ Multivariable Model 1 included age, sex, BMI, CKD stage, and comorbidities (DM, CVD, and anemia). ^4^ Multivariable Model 2 included Model 1 plus alcohol drinking status, smoking status, blood biochemistry (phosphate, albumin, CRP, and BNP), drugs (ESA, anti-hyperuricemia, phosphate binders, anti-hyperlipidemia, and RASi), systolic blood pressure, LVEF, total-body skeletal muscle mass, urinary protein excretion, urinary sodium excretion, and max-IMT. ^5^ Multivariable Model 3 included multiple imputation for missing data (24-h urine collection: *n* = 21) for sensitivity analysis, and it was adjusted by factors in Model 2. Numbers of patients with very low protein intake (VLPI), low protein intake (LPI), and moderate protein intake (MPI) were 65, 158, and 119, respectively. ^6^ To estimate association between protein intake and age, age-stratified (≤65 and >65 years) models [27] were used. ^7^ End-stage renal disease included hemodialysis, peritoneal dialysis, and kidney transplantation.

**Table 3:** Mean change in renal function and other outcomes per year according to dietary protein intake ^1^.

	VLPI		LPI		MPI		*p* Value ^2^
**Change in eGFR** **(mL/min/1.73 m^2^/year)**							
All	0.84	(−3.69 to 5.37)	−0.23	(−4.80 to 4.34)	−0.97	(−5.63 to 3.70)	**0.032**
≤65 years	−0.34	(−8.37 to 7.69)	−1.34	(−10.15 to 7.47)	−1.88	(−10.85 to 7.09)	0.812
>65 years	1.06	(−4.24 to 6.36)	−0.69	(−6.02 to 4.63)	−0.98	(−6.46 to 4.49)	**0.028**
**Change in spot urine protein** **(g/gCr/year)**							
All	−0.87	(−2.36 to 0.62)	−0.36	(−1.87 to 1.14)	−0.58	(−2.12 to 0.95)	0.787
≤65 years	−0.81	(−3.38 to 1.76)	−0.15	(−2.97 to 2.66)	−1.31	(−4.18 to 1.56)	0.338
>65 years	−0.82	(−2.61 to 0.97)	−0.37	(−2.16 to 1.42)	−0.28	(−2.13 to 1.56)	0.375
**Change in body weight** **(%/year)**							
All	1.70	(−4.08 to 7.49)	2.74	(−3.14 to 8.61)	1.54	(−4.47 to 7.54)	0.312
≤65 years	−7.79	(−23.37 to 7.80)	−7.75	(−24.00 to 8.50)	−7.44	(−23.66 to 8.78)	0.986
>65 years	2.54	(−3.99 to 9.06)	3.89	(−2.71 to 10.48)	2.66	(−4.19 to 9.50)	0.308
**Change in serum albumin (g/dL/year)**							
All	0.08	(-0.56 to 0.71)	0.15	(−0.50 to 0.79)	0.25	(−0.41 to 0.91)	0.109
≤65 years	−1.00	(−1.67 to −0.33)	−0.61	(−1.34 to 0.13)	−0.56	(−1.31 to 0.19)	**0.048**
>65 years	0.64	(−0.20 to 1.48)	0.64	(−0.21 to 1.49)	0.73	(−0.14 to 1.60)	0.338
**Change in serum phosphate (mg/dL/year)**							
All	−0.08	(−0.71 to 0.55)	0.04	(−0.59 to 0.68)	0.01	(−0.63 to 0.66)	0.804
≤65 years	0.34	(−0.82 to 1.50)	0.41	(−0.86 to 1.68)	0.20	(−1.09 to 1.50)	0.396
>65 years	−0.13	(−0.88 to 0.61)	0.05	(−0.70 to 0.80)	0.08	(−0.69 to 0.85)	0.283

^1^ All values are mean change in outcomes per year (95% confidence interval). Mean differences were evaluated by analysis of covariance (ANCOVA) adjusted for age, sex, BMI, CKD stage, comorbidities (DM, CVD, and anemia), alcohol drinking status, smoking status, blood biochemistry (phosphate, albumin, CRP, and BNP), drugs (ESA, anti-hyperuricemia, phosphate binders, anti-hyperlipidemia, and RASi), systolic blood pressure, LVEF, total-body skeletal muscle mass, urinary protein excretion, urinary sodium excretion, and max-IMT. Bold values are statistically significant (*p* < 0.05). ^2^ *p*-linear trend was calculated by using the treatment exposure as a continuous variable.

## Supplementary Materials

The following are available online at http://www.mdpi.com/2072-6643/10/11/1744/s1, Table S1: Protein and sodium intake from two 24-h urine samples, coefficients of variation, and within-patient to between-patient variance ratios shown per sex.; Table S2: Number of days and number of patients required to ensure accuracy in the group’s and individual patients’ usual (“true”) mean intake based on 24-h urine collection, shown per sex.

## References

[1] O’Hare A.M., Bertenthal D., Covinsky K.E., Landefeld C.S., Sen S., Mehta K., Steinman M.A., Borzecki A., Walter L.C. (2006). Mortality risk stratification in chronic kidney disease: One size for all ages?. J. Am. Soc. Nephrol..

[2] Lin M.Y., Chiu Y.W., Lee C.H., Yu H.Y., Chen H.C., Wu M.T., Hwang S.J. (2013). Factors associated with CKD in the elderly and nonelderly population. Clin. J. Am. Soc. Nephrol..

[3] Anderson S., Halter J.B., Hazzard W.R., Himmelfarb J., Horne F.M., Kaysen G.A., Kusek J.W., Nayfield S.G., Schmader K., Tian Y. (2009). Prediction, progression, and outcomes of chronic kidney disease in older adults. J. Am. Soc. Nephrol..

[4] O’Hare A.M., Choi A.I., Bertenthal D., Bacchetti P., Garg A.X., Kaufman J.S., Walter L.C., Mehta K.M., Steinman M.A., Allon M. (2007). Age affects outcomes in chronic kidney disease. J. Am. Soc. Nephrol..

[5] Kovesdy C.P., Kopple J.D., Kalantar-Zadeh K. (2013). Management of protein-energy wasting in non-dialysis-dependent chronic kidney disease: Reconciling low protein intake with nutritional therapy. Am. J. Clin. Nutr..

[6] Fouque D., Kalantar-Zadeh K., Kopple J., Cano N., Chauveau P., Cuppari L., Franch H., Guarnieri G., Ikizler T.A., Kaysen G. (2008). A proposed nomenclature and diagnostic criteria for protein-energy wasting in acute and chronic kidney disease. Kidney Int..

[7] Fouque D., Pelletier S., Mafra D., Chauveau P. (2011). Nutrition and chronic kidney disease. Kidney Int..

[8] Ikizler T.A., Cano N.J., Franch H., Fouque D., Himmelfarb J., Kalantar-Zadeh K., Kuhlmann M.K., Stenvinkel P., TerWee P., Teta D. (2013). Prevention and treatment of protein energy wasting in chronic kidney disease patients: A consensus statement by the International Society of Renal Nutrition and Metabolism. Kidney Int..

[9] Inker L.A., Astor B.C., Fox C.H., Isakova T., Lash J.P., Peralta C.A., Kurella Tamura M., Feldman H.I. (2014). KDOQI US commentary on the 2012 KDIGO clinical practice guideline for the evaluation and management of CKD. Am. J. Kidney Dis..

[10] Kalantar-Zadeh K., Fouque D. (2017). Nutritional management of chronic kidney disease. N. Engl. J. Med..

[11] Iseki K., Yamagata K. (2016). A practical approach of salt and protein restriction for CKD patients in Japan. BMC Nephrol..

[12] Giovannetti S., Maggiore Q. (1964). A low-nitrogen diet with proteins of high biological value for severe chronic uraemia. Lancet.

[13] Fouque D., Aparicio M. (2007). Eleven reasons to control the protein intake of patients with chronic kidney disease. Nat. Clin. Pract. Nephrol..

[14] Fouque D., Laville M. (2009). Low protein diets for chronic kidney disease in non diabetic adults. Cochrane Database Syst. Rev..

[15] Levey A.S., Greene T., Sarnak M.J., Wang X., Beck G.J., Kusek J.W., Collins A.J., Kopple J.D. (2006). Effect of dietary protein restriction on the progression of kidney disease: Long-term follow-up of the Modification of Diet in Renal Disease (MDRD) Study. Am. J. Kidney Dis..

[16] Cianciaruso B., Pota A., Bellizzi V., Di Giuseppe D., Di Micco L., Minutolo R., Pisani A., Sabbatini M., Ravani P. (2009). Effect of a low-versus moderate-protein diet on progression of CKD: Follow-up of a randomized controlled trial. Am. J. Kidney Dis..

[17] Halbesma N., Bakker S.J., Jansen D.F., Stolk R.P., De Zeeuw D., De Jong P.E., Gansevoort R.T., Group P.S. (2009). High protein intake associates with cardiovascular events but not with loss of renal function. J. Am. Soc. Nephrol..

[18] Rand W.M., Pellett P.L., Young V.R. (2003). Meta-analysis of nitrogen balance studies for estimating protein requirements in healthy adults. Am. J. Clin. Nutr..

[19] Campbell W.W., Crim M.C., Dallal G.E., Young V.R., Evans W.J. (1994). Increased protein requirements in elderly people: New data and retrospective reassessments. Am. J. Clin. Nutr..

[20] Von Elm E., Altman D.G., Egger M., Pocock S.J., Gotzsche P.C., Vandenbroucke J.P., Initiative S. (2007). The Strengthening the Reporting of Observational Studies in Epidemiology (STROBE) statement: Guidelines for reporting observational studies. PLoS Med..

[21] Wang Z.M., Gallagher D., Nelson M.E., Matthews D.E., Heymsfield S.B. (1996). Total-body skeletal muscle mass: Evaluation of 24-h urinary creatinine excretion by computerized axial tomography. Am. J. Clin. Nutr..

[22] Matsuo S., Imai E., Horio M., Yasuda Y., Tomita K., Nitta K., Yamagata K., Tomino Y., Yokoyama H., Hishida A. (2009). Revised equations for estimated GFR from serum creatinine in Japan. Am. J. Kidney Dis..

[23] Maroni B.J., Steinman T.I., Mitch W.E. (1985). A method for estimating nitrogen intake of patients with chronic renal failure. Kidney Int..

[24] Masud T., Manatunga A., Cotsonis G., Mitch W.E. (2002). The precision of estimating protein intake of patients with chronic renal failure. Kidney Int..

[25] Fukumoto A., Asakura K., Murakami K., Sasaki S., Okubo H., Hirota N., Notsu A., Todoriki H., Miura A., Fukui M. (2013). Within- and between-individual variation in energy and nutrient intake in Japanese adults: Effect of age and sex differences on group size and number of records required for adequate dietary assessment. J. Epidemiol..

[26] Hsu J.Y., Roy J.A., Xie D., Yang W., Shou H., Anderson A.H., Landis J.R., Jepson C., Wolf M., Isakova T. (2017). Statistical methods for cohort studies of CKD: Survival analysis in the setting of competing risks. Clin. J. Am. Soc. Nephrol..

[27] Levine M.E., Suarez J.A., Brandhorst S., Balasubramanian P., Cheng C.W., Madia F., Fontana L., Mirisola M.G., Guevara-Aguirre J., Wan J. (2014). Low protein intake is associated with a major reduction in IGF-1, cancer, and overall mortality in the 65 and younger but not older population. Cell. Metab..

[28] Buuren S.V., Groothuis-Oudshoorn K. (2011). Mice: Multivariate Imputation by Chained Equations in R. J. Stat. Softw..

[29] Rhee C.M., Ahmadi S.F., Kovesdy C.P., Kalantar-Zadeh K. (2018). Low-protein diet for conservative management of chronic kidney disease: A systematic review and meta-analysis of controlled trials. J. Cachexia Sarcopenia Muscle.

[30] Shinaberger C.S., Kilpatrick R.D., Regidor D.L., McAllister C.J., Greenland S., Kopple J.D., Kalantar-Zadeh K. (2006). Longitudinal associations between dietary protein intake and survival in hemodialysis patients. Am. J. Kidney Dis..

[31] Giordano M., Ciarambino T., Castellino P., Paolisso G. (2013). Light and shadows of dietary protein restriction in elderly with chronic kidney disease. Nutrition.

[32] Isoyama N., Qureshi A.R., Avesani C.M., Lindholm B., Barany P., Heimburger O., Cederholm T., Stenvinkel P., Carrero J.J. (2014). Comparative associations of muscle mass and muscle strength with mortality in dialysis patients. Clin. J. Am. Soc. Nephrol..

[33] Stenvinkel P., Carrero J.J., Von Walden F., Ikizler T.A., Nader G.A. (2016). Muscle wasting in end-stage renal disease promulgates premature death: Established, emerging and potential novel treatment strategies. Nephrol. Dial. Transplant..

[34] Jesudason D.R., Pedersen E., Clifton P.M. (2013). Weight-loss diets in people with type 2 diabetes and renal disease: A randomized controlled trial of the effect of different dietary protein amounts. Am. J. Clin. Nutr..

[35] Knight E.L., Stampfer M.J., Hankinson S.E., Spiegelman D., Curhan G.C. (2003). The impact of protein intake on renal function decline in women with normal renal function or mild renal insufficiency. Ann. Intern. Med..

[36] Coresh J., Turin T.C., Matsushita K., Sang Y., Ballew S.H., Appel L.J., Arima H., Chadban S.J., Cirillo M., Djurdjev O. (2014). Decline in estimated glomerular filtration rate and subsequent risk of end-stage renal disease and mortality. JAMA.

[37] Matsushita K., Chen J., Sang Y., Ballew S.H., Shimazaki R., Fukagawa M., Imai E., Coresh J., Hishida A. (2016). Risk of end-stage renal disease in Japanese patients with chronic kidney disease increases proportionately to decline in estimated glomerular filtration rate. Kidney Int..

[38] Iff S., Wong G., Webster A.C., Flood V., Wang J.J., Mitchell P., Craig J.C. (2014). Relative energy balance, CKD, and risk of cardiovascular and all-cause mortality. Am. J. Kidney Dis..

[39] Cederholm T., Jensen G.L., Correia M., Gonzalez M.C., Fukushima R., Higashiguchi T., Baptista G., Barazzoni R., Blaauw R., Coats A. (2018). GLIM criteria for the diagnosis of malnutrition-A consensus report from the global clinical nutrition community. Clin. Nutr..

[40] Hall K.D., Sacks G., Chandramohan D., Chow C.C., Wang Y.C., Gortmaker S.L., Swinburn B.A. (2011). Quantification of the effect of energy imbalance on bodyweight. Lancet.

[41] Lew Q.J., Jafar T.H., Koh H.W., Jin A., Chow K.Y., Yuan J.M., Koh W.P. (2017). Red Meat Intake and Risk of ESRD. J. Am. Soc. Nephrol..

