# Supplementary material for: Age Modifies the Association of Dietary Protein Intake with All-Cause Mortality in Patients with Chronic Kidney Disease
Source: Nutrients. 2018 Nov 13;10(11):1744. doi: 10.3390/nu10111744 (PMC6265861; doi:10.3390/nu10111744)
Supplement: Supplementary file 1 [file nutrients-10-01744-s001.pdf]

Supplemental Table 1 Protein and sodium intake from two 24-h urine samples, coefficients of variation, and within-patient to between-patient variance ratios shown per sex.

	First Sample		Second Sample		CV_w (%)	CV_b (%)	VR
	Mean	SD	Mean	SD			
Male patients (*n* = 86)							
Protein intake (g/day)	36.7	9.6	35.4	8.5	1.57	1.80	0.87
Sodium intake (mmol/day)	64.8	20.9	51.1	17.3	4.97	7.95	0.62
Female patients (*n* = 204)							
Protein intake (g/day)	45.8	11.8	43.9	10.9	1.83	2.26	0.81
Sodium intake (mmol/day)	74.3	27.5	55.0	21.5	5.33	10.31	0.52

CV_b, coefficient of between-patient variation; CV_w, coefficient of within-patient variation; SD, standard deviation; VR, ratio of within- to between-patient variance.

Supplemental Table 2 Number of days and number of patients required to ensure accuracy in the group's and individual patients' usual ("true") mean intake based on 24-h urine collection, shown per sex 1.

	Male Patients		Female Patients	
	Protein Intake	Sodium Intake	Protein Intake	Sodium Intake
Specified % deviation [2]				
2.5	4	54	5	83
5	1	14	1	21
10	0	3	0	5
20	0	1	0	1
Specified correlation coefficient [3]				
0.80	2	1	1	1
0.85	2	2	2	1
0.90	4	3	3	2
0.95	8	6	7	5
Specified % deviation [4]				
5	0	4	1	4
10	0	1	0	1
20	0	0	0	0
30	0	0	0	0

[1] Number of patients or number of days is shown. When a value was less than 1 day or 1 patient, it was rounded to the nearest whole number. CV_b, coefficient of between-patient variation; CV_w, coefficient of within-patient variation; VR, ratio of within- to between-patient variance. [2] Group size for 24-h urine collection assuming a single observation for each patient = $1.96^2 \times [(CV_b^2 + CV_w^2) / D_0^2]$, where D_0 = the specified % deviation of the group mean intake from the group usual ("true") mean intake. [3] Number of days of 24-h urine collection needed for estimation of specified level of correlation coefficient = $[r^2 / (1 - r^2)] \times VR$, where r = unobservable correlation coefficient between observed and usual ("true") mean intake of patients and VR = within-patient/between-patient variance ratio (CV_w / CV_b). [4] Number of days needed for estimation of patient's "true" mean intake with 95% CI within the specified % deviation required for 24-h urine collection = $(1.96 \times CV_w / D_1)^2$, where D_1 = the specified % deviation of patient mean from the usual ("true") mean intake.
